# Order and Complexity in the RNA World

**DOI:** 10.3390/life13030603

**Published:** 2023-02-21

**Authors:** Christian Mayer

**Affiliations:** Institute of Physical Chemistry, CENIDE, University of Duisburg-Essen, 45141 Essen, Germany; christian.mayer@uni-due.de; Tel.: +49-201-183-2570

**Keywords:** RNA world, order, complexity, origin of life, molecular evolution, prebiotic chemistry, statistical thermodynamics, entropy

## Abstract

The basic idea of the RNA world as an early step towards life relies on a molecular evolution process based on self-replicating RNA strands. It is probably the oldest and most convincing model for efficient prebiotic evolution. Obviously, the functionality of RNA sequences depends on order (i.e., the definition of their sequence) as well as on complexity (i.e., the length of their sequence). Order and complexity seem to be crucial parameters in the course of RNA evolution. In the following, an attempt is made to define these parameters and to identify characteristic mechanisms of their development. Using a general RNA world scenario including the free monomer units, the sequential order is defined based on statistical thermodynamics. The complexity, on the other hand, is determined by the size of a minimal algorithm fully describing the system. Under these conditions, a diagonal line in an order/complexity-diagram represents the progress of molecular evolution. Elementary steps such as repeated random polymerization and selection follow characteristic pathways and finally add up to a state of high system functionality. Furthermore, the model yields a thermodynamic perspective on molecular evolution, as the development of a defined polymer sequence has a distinct influence on the entropy of the overall system.

## 1. Defining Progress in an RNA World

The important role of RNA as the key polymer in the origin of life was initially proposed in the sixties of the last century [1,2,3]. It was driven by the discovery that RNA strands can have significant catalytic activity [4,5], including the phenomenon that they may facilitate their own reproduction. This opens the door to an efficient RNA-based self-sustaining molecular evolution, the RNA world [6]. Since its proclamation in 1986, this model has played a dominating role in the general discussion about prebiotic evolution [7,8,9,10,11,12,13,14].

According to the general understanding, the evolution of RNA starts with some random polymerization of different (proto-)nucleotides, e.g., containing the four bases adenine, guanine, cytosine and uracil (or corresponding predecessors [15]). Hereby, a large number of accidental sequences are formed, a certain fraction of which may have catalytic functions. In some cases, they may catalyze, directly or indirectly, their own reproduction, which eventually will lead to a selection and accumulation of these distinct RNA sequences. As originally proposed by Walter Gilbert, the RNA molecules may “evolve in self-replicating patterns, using recombination and mutation to explore new functions and to adapt to new niches” [6]. In the course of this evolution process, the functionality of the selected RNA species will grow, connected to improved catalytic functions and increased efficiency of the reproduction cycle. Finally, the selected species will dominate the polymerized RNA fraction in the system.

Following this general idea, one may imagine an idealized primordial soup at a given stage of the evolution process where nucleotides occur next to their oligomers and polymers in a homogeneous solution. Is there a possible way to rate this given state regarding the overall process of evolution? Of course, one could determine the catalytic activities of some of the given species, but based on these data, it would be very complicated to obtain access to an overall picture. Instead, it makes sense to focus on more general system parameters, such as order and complexity [16,17].

**Figure 1:**
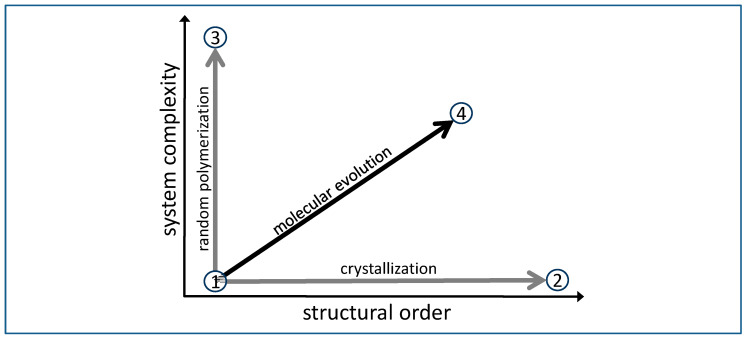
Diagram showing four characteristic states in an RNA world scenario. ① Mix of nucleotides: low order, low complexity. ② Crystals formed by nucleotides: high order, low complexity. ③ Random polymer formed by nucleotides: low order, high complexity. ④ Product of an evolution process: high order (many chains with common defined sequences), high complexity (length of the sequence) and, correspondingly, high functionality.

The principle approach of order and complexity in case of RNA-based molecular evolution is roughly illustrated in Figure 1. The process starts with a mixed solution of monomer units that is low in order and low in complexity, therefore assigned to point 1 near the lower left corner of the diagram. Increasing the order of the system is quite simple. As an example, one may bring the system into a dry state, hereby forcing crystallization. Complexity remains more or less constant, therefore, the process follows a horizontal line. Point 2 in the diagram represents monomer crystals with a strongly increased order, but of course no functionality, since the system lacks sufficient complexity.

Increasing complexity, on the other hand, is quite easy as well. Inducing random polymerization under suitable conditions may finally lead to a single polymer chain (point 3). The long random sequence of this single chain represents a very complex situation, but practically no progress in terms of order. As a random chain, it will most likely not support any function.

Functionality such as catalysis needs a high degree of complexity (a certain length of the chain) *combined* with a high degree of order (many chains with identical, well-defined sequences). Such a combination (e.g., point 4 in Figure 1) cannot be reached in a single move. Instead, it requires many subsequent steps in a process of molecular evolution. In an experimental reproduction of an evolution process, the development towards order and complexity may be followed if the monomer composition, chain lengths and chain sequences are monitored over time and if possible interactions between the system components are considered. A basic approach for the determination of order and complexity from these data is proposed in the following sections.

## 2. A “Random Walk” through the System

**Figure 2:**
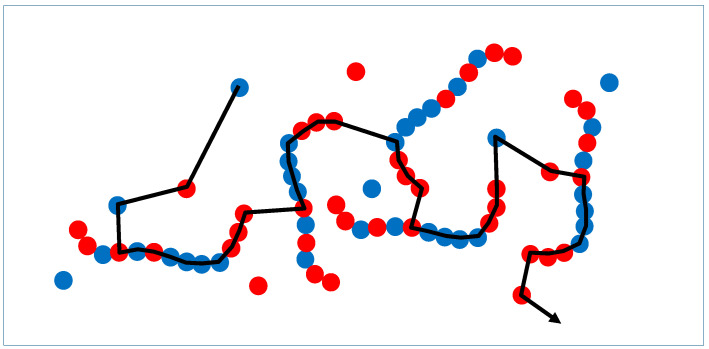
Schematic representation of a fraction of a random walk following rules 1–5 through a system with monomer units (separate circles) and repetitive units in oligomer chains.

In order to give those key parameters (order and complexity) a clear basis, they need a proper definition that, in turn, requires a simplified model approach to the system. This model is just meant to monitor the state of the system at a given point in time and does not represent a molecular process. As a starting point, we remove all solvent molecules as well as those components that do not take part in the course of the evolution. Looking at the remaining monomer units and chain molecules, we implement something similar to a random walk from monomer to monomer, from chain unit to chain unit and in between chains and monomers (Figure 2). Disregarding the difference between chain units and monomers, the term “unit” will be used in the following. The pathway through the system is not fully random, but follows a given set of rules. It may resemble a classical random walk, but does not include statistical decisions for the most part. Actually, at one point in time, it is exclusively random in terms of the starting point. The rules are as follows:

(1) The starting point may be any unit of the system.
(2) In each step, the move preferably occurs to the closest adjacent unit (nearest neighbor).
(3) If rule 2 leads to a unit that is already part of the given path, choose the one with the next-shortest distance. This rule applies repeatedly until the next step is leading to a “fresh” unit that has not been part of the path so far.
(4) The random walk stops after *N* steps, with *N* being the total number of units in the system.
(5) The random walk is repeated again and again over a certain time window to account for dynamic reorientation processes (molecular dynamics) in the system.

These rules guarantee that the resulting set of pathways reflects a characteristic cross-section of the system. Rule no. 1 allows for the definition of *N* different pathways until the full system is specified. Rule no. 2 accounts for contacts and nearest neighborhoods resulting from covalent bonds in a chain molecule as well as brief contacts between molecules (as formed by hydrogen bonds in a base-pairing step) or temporary structures formed inside membrane bilayers. Rule no. 3 efficiently prevents the formation of closed loops. Of course, the random walks will vary over time, as the system undergoes dynamic changes. The time window of the random walks according to rule no. 5 is chosen such that molecular dynamics are possible, while no chemical structures are changed. Under these conditions, the full set of pathways will represent a complete image of the system’s intermolecular interactions. It will account for preferred intermolecular contacts such as base pairings, polymer entanglements, intermediate structures in reduplication processes etc. There may be alternative concepts allowing for structural monitoring of the system, but the described procedure should be among the most efficient strategies.

## 3. Defining the Order of the System—Reciprocal Sequential Entropy

As a manifestation of the system’s order, we will use the reciprocal entropy assigned to the entity of all random walks. Following the rules of statistical thermodynamics, one can derive a general expression for this characteristic “sequential” entropy contribution of a mixture of monomer and oligomer chains based on a set of system parameters:

(1) The number *n* of different types of monomer units (e.g., four in case of conventional RNA).
(2) The relative contributions *r_i_* of all given monomer types *i* in solution (with *r*_1_ + *r*_2_ + *r*_3_ + …+ *r_n_* = 1). In case of RNA and for equal concentrations of all bases, we obtain *r*_1_ = *r*_2_ = *r*_3_ = *r*_4_ = 0.25.
(3) The average number of monomers *M* between two chains in the random walk (in number of units).
(4) The average chain length *L* (in number of units).
(5) The predictability *p_k_* of a given chain unit in accordance with a (partially) defined chain sequence. Note that the index “*k*” generally does not coincide with the index “*i*”. Instead, *k* = 1 denotes the most likely unit to follow, *k* = 2 the second-most likely, etc. Completely defined chains, therefore, lead to *p*_1_ = 1 and *p*_2_ = *p*_3_ = *p*_4_ = … = *p_n_* = 0), completely random chains to *p*_1_ = *p*_2_ = *p*_3_ = … = *p_n_* = 1/*n*. The values for *p_k_* are a measure for the average degree of definition of the chain sequences.
(6) The relative accessibility *a_j_* of unit j of the chains in solution (with *a_j_* = 0 for a completely inaccessible segment, and *a*_1_ + *a*_2_ + *a*_3_ + … + *a_L_* = 1). This parameter set accounts for the average chain conformation and its preferred contact sites with monomer units in solution.
(7) The total number *N* of units in the system and on the pathway of the random walk.

With these parameters given, the characteristic entropy contribution *S_r_* (the sequential entropy contribution) of a random walk is derived as (for a justification of Equation (1), please see Supporting Information):

(1) $$\begin{array}{c} S_{r}=k\;\ln\;w=k\;M\;\left(\frac{N}{M+\frac{L}{2}}\right)\sum_{i=1}^{n}r_{i}\ln(1/r_{i})\;+\\ k\;\left(\frac{N}{M+\frac{L}{2}}\right)\sum_{j=1}^{L}a_{j}\ln(1/a_{j})\;+\\ k\;\frac{L}{2}\;\left(\frac{N}{M+\frac{L}{2}}\right)\sum_{k=1}^{n}p_{k}\ln(1/p_{k}) \end{array}$$

with k as Boltzmann’s constant and *w* as the statistical weight defined by the variety within the set of random walks. If we assume that a certain fraction *p* of the chains is random (with *p*_1_ = *p*_2_ = *p*_3_ = … = *p_n_* = 1/*n*) and the fraction (1 − *p*) has fully defined sequences resulting from efficient selection (with *p*_1_ = 1 and *p*_2_ = *p*_3_ = *p*_4_ = … = *p_n_* = 0), Equation (1) turns into

(2) $$\begin{array}{c} S_{r}=k\;\ln\;w=k\;M\left(\frac{N}{M+\frac{L}{2}}\right)\sum_{i=1}^{n}r_{i}\ln(1/r_{i})\;+\\ k\;\left(\frac{N}{M+\frac{L}{2}}\right)\sum_{j=1}^{L}a_{j}\ln(1/a_{j})\;+\\ k\;p\;\frac{L}{2}\;\left(\frac{N}{M+\frac{L}{2}}\right)\ln n \end{array}$$

For equal amounts of all monomer types *i* in the solution (*r*_1_ = *r*_2_ = *r*_3_ = … = *r_n_* = 1/*n*) and for equal relative accessibilities within the defined chains (*a*_1_ = *a*_2_ = *a*_3_ = … = *a_L_* = 1/*L*), Equation (2) further simplifies to:

(3) $$\begin{array}{c} S_{r}=k\;\ln\;w=k\;M\left(\frac{N}{M+\frac{L}{2}}\right)\ln\;n\;+\\ k\;\left(\frac{N}{M+\frac{L}{2}}\right)\ln\;L\;+\\ k\;p\frac{L}{2}\;\left(\frac{N}{M+\frac{L}{2}}\right)\ln\;n \end{array}$$

The applicability of Equation (3) may be shown on four characteristic cases that correspond to points 1 to 4 in Figure 1. (all for equal monomer contributions and relative accessibilities):

(1) **Pure monomer**. If no chains are present and the system consists of monomer units only, the average chain length is *L* = 1 and all units are fully random with *p* = 1. In this case, Equation (3) reduces to *S_r_* = k *N* ln *n.* For four different monomer varieties, the corresponding entropy contribution for one mol of units (*N* = 6.022 ∙ 10^23^) amounts to *S_r_* = 11.526 J/(K∙mol), which is equivalent to the mixing entropy of the four different units (with 0.25 mol of each monomer) in the same overall volume. This leads to a relatively large entropy contribution *S_r_* and, correspondingly, to a small sequential order 1/*S_r_*; this situation is represented by point 1 in Figure 1.
(2) **Crystals**. If we induce crystallization, e.g., from a mixed monomer solution by removal of the solvent, we expect to obtain separate crystals of pure monomer types 1, 2, 3, …, *n* next to each other. In the random walk model, each crystal is the equivalent to a very long chain (with a very large length *L*), while no monomer is present (*M* = 0). At the same time, we have to consider *n* = 1 for a dominating part of the random walk, since only one single type of monomer is found within each individual crystal. This given, Equation (3) simplifies to *S_r_* = k (2*N*/*L*) ln *L*, an entropy term which reflects the variability of the possible contact points between the different crystals in the course of the random walk. In effect, this leads to a very low sequential entropy *S_r_* and, correspondingly, to a high degree of order given by 1/*S_r_*. In Figure 1, this situation could be assigned to point 2 (Figure 1).
(3) **Random chains, no monomer**. In case of random chains (*p* = 1) in complete absence of all monomers (meaning *M* = 0), Equation (3) turns into *S_r_* = k (2*N*/*L*) ln *L* + k *N* ln *n*, a term largely dominated the mixing entropy of the *N* units, as all of them are random over the full pathway. Due to the variability of the chain contacts, the entropy contribution is slightly larger than in case 1 and again leads to a slightly lower sequential order 1/*S_r_*. This situation is generally referred to as the asphalt problem [18] and would correspond to point 3 (Figure 1).
(4) **Defined chains, no monomer**. In a system without any residual monomer (which means that *M* = 0) and fully defined chains with common sequences (*p* = 0), Equation (3) reduces to *S_r_* = k (2*N*/*L)* ln *L*, an entropy term similar to the one in case 3, but this time reflecting the possible contact points between the chains in the course of the random walk. Depending on the average chain length *L*, the resulting entropy contribution *S_r_* is significantly smaller than in case 1. In Figure 1, this situation reflects the product of an extremely successful evolution with a high sequential order 1/*S_r_* as indicated by point 4 (Figure 1).

An interesting fact results from a comparison between cases 3 and 4, random chains vs. defined chains. The difference between both entropy contributions is given by Δ*S_r_* = k *N* ln *n*, equivalent to the monomer mixing entropy which is in the range of several J/K for one mol of chain units. This value is of clear thermodynamic relevance. It describes the driving force for the loss of well-defined chain sequences and is not necessarily connected to the formation of shorter chains. If we describe the defined chains as “living” and the random chains as “dead”, this value represents the amount of entropy separating these two states. Of course, it is simply based on the low statistical weight of the defined chain sequence that in the ideal case is equal to unity.

It is important to note that these results account for the statistics of all possible random walk pathways. They also include possible intermolecular interactions between chains, for example, during the chain reproduction process. In this case, the variable *M*, the number of monomer units between chains on the pathway decreases based on preferred chain-to-chain contacts. This is especially relevant in cases where evolution occurs under the influence of wet–dry cycles [19,20]. Base pairing effects can be introduced by using Equation (1) instead of Equation (2) or (3) and increasing the probability *p_i_* for a specific type of monomer and for a specific chain site above its relative occurrence in the solution. If chain molecules accumulate locally, e.g., inside lipid bilayer structures [21,22], this again leads to a corresponding reduction of the parameter *M* and changes in the specific accessibilities a*_j_*. If a combination of wet–dry cycling and membrane structure formation occurs, such as in the hydrothermal pool scenario [23], the approach can be adapted accordingly. Finally, the description can be designed to describe the influence of solid surfaces in the system [24,25]. Overall, the given approach of using the random walk together with the statistical description by Equation (1) is very versatile and accounts for all possible states during an RNA evolution. It delivers the sequential entropy contribution *S_r_* and, with its reciprocal value 1/*S_r_*, an important measure for the sequential order in the system. The value for *S_r_* actually coincides with the system entropy proposed in a machine learning approach to evolution [26,27,28]. In this context, a spontaneous decrease of sequential entropy, e.g., during a successful reduplication process under dissipation of energy, is assigned to the entropy decrease connected to the second law of learning [26].

## 4. Defining the Complexity of the System—The Size of the Reproducing Algorithm

Among the many approaches to determine system complexity as a parameter, the idea originally developed by Andrey Nikolaevich Kolmogorov seems to be most appropriate to characterize prebiotic development [29,30,31,32]. It is based on the assumption that, for every structure, there is a minimal size of an algorithm (or computer program) which fully describes its entity and all of its details. The size of this computer algorithm in a universal description language in bit or byte may serve as a measure for the degree of complexity of the system. Even though it is hard or even impossible to determine the minimal size of the algorithm exactly (a problem known as Chaitin’s incompleteness theorem [33]), this number still can be approached and estimated for a given system.

In connection with the “random walk” described in Section 2, it means one has to find an algorithm that, if run for an infinite number of times, leads to a set of results identical to that of the real system if sampled by an indefinite number of random walks over time. The length of the code of this algorithm (in bit or byte) determines the given degree of complexity. As an example, the complexity *c* of a defined chain sequence is given by the number of bit necessary for its description. For a chain formed by *L* units with *n* = 4 (as for RNA), it amounts to *c* = 2*L* (in bit) as 2 bit are needed to define each unit. In general, for a choice from *n* types of units, we obtain

*c* = *L* log_2_
*n* (in bit) (4)

For undefined, random chains of length *L*, the situation turns out to be more complicated. In principle, there are two strategies to account for the different chain sequences in the system:

(a) The algorithm could create a list of all *n^L^* possible permutations for a chain of a length *L*, and then assign an integer number between 0 and *N*/*L*, the upper limit defined by the average number of chains in the total system. With a large number of possible permutations, these values would require the largest part of the code. Therefore, the value for the complexity can be approximated by:
  (5) $$c_{1}\approx n^{L}\log_{2}\;\frac{N}{L}\;(\mathrm{in}\;\mathrm{bit})$$
  The precise value for c could be larger, as the short code for the creation of the list of permutations must be accounted for, but it could also be smaller, as many zeros or small values on the list of integers could be compressed in an optimized coding.
(b) The algorithm could contain a list of every single sequence of every single chain in the system. Since all chains would add up to an overall length of N, the complexity could then be approximated by:
  *c*_2_*N* log_2_
*n* (in bit) (6)

While strategy (a) is more efficient for short chains, strategy (b) definitely would be more suitable for long chains. According to the definition of the Kolmogorov complexity, it is the smaller value of the set *c_1_* and *c_2_* that defines the degree of complexity. In borderline cases, it may be even adequate to combine both strategies in order to achieve the smallest code. In case of a wide distribution of chain lengths, the strategy (a) may account for the shorter chains, while strategy (b) may be chosen for the longer chains.

If we assume that a certain fraction *p* of the chains is random (with *p*_1_ = *p*_2_ = *p*_3_ = … = *p_n_* = 1/*n*) and that the fraction (1 − *p*) has a fully defined sequence (with *p*_1_ = 1 and *p*_2_ = *p*_3_ = *p*_4_ = … = *p_n_* = 0), the information on the defined chain (Equation (4)) needs to be combined with the information of the random chains with the fraction *p*. In this case, we either obtain

(7) $$c_{1}\approx L\;\log_{2}\;n+n^{L}\log_{2}\;\frac{pN}{L}\;(\mathrm{in}\;\mathrm{bit})$$

or, alternatively

*c_2_* ≈ *L* log_2_
*n* + *pN* log_2_
*n* (in bit) (8)

As a last step, we may finally account for the monomer content in the solution. The value of *M* as the average number of monomer units in between the chains principally reduces the effort to define the random chain sequences. The number of random chains *pN*/*L* in Equations (7) and (8) reduces to to *pN*/(*L* + *M*). Consequently, we obtain

(9) $$c_{1}\approx L\;\log_{2}\;n+n^{L}\log_{2}\;\frac{pN}{L+M}\;(\mathrm{in}\;\mathrm{bit})$$

or, alternatively

(10) $$c_{2}\approx L\;\log_{2}\;n+L\;\frac{pN}{L+M}\;\log_{2}\;n\;(\mathrm{in}\;\mathrm{bit})$$

as the terms that potentially determine the system’s complexity, depending on which one is smaller.

Just as for the degrees of order, we derive the resulting degrees of complexity for the characteristic cases indicated in Figure 1:

(1) **Pure monomer**. We assume that no chains are present and the system consists of monomer units of equal relative contributions (*r*_1_ = *r*_2_ = *r*_3_ = … = *r_n_* = 1/*n*). Under these circumstances, the choice of the next monomer in the random walk is fully reproduced by a suitable random number generator that produces the integers 1, 2, 3, …, *n* at equal probability. This random number generator could be a subroutine that would be called repetitively in a loop for *N* times. If run repeatedly for an infinite number of times, this program would fully reproduce the statistics of a corresponding infinite number of consecutive random walks. The overall size of the program code could be limited to a few byte, corresponding to a very low degree of complexity. This situation is represented by point 1 in Figure 1.
(2) **Crystals**. If we induce crystallization, e.g., from a mixed monomer solution by removal of the solvent, we expect to obtain separate crystals of pure monomer types 1, 2, 3, … n next to each other. Simulating the random walk, the program would run the random number generator once to define the type *i* of the starting crystal. It would then assume a random walk through the same units *i* for an average length determined by the average size of the crystals. After that, the type of the following crystal *i’* would be determined by the random generator, and so on, until the full total length *N* is achieved. Again, if this routine is repeated for a very large number of times, the statistics of the results would be identical to the one for random walks in the real system. The size of the program code would only be slightly larger than in case 1, so the complexity of this situation is still very low. In Figure 1, it could be assigned to point 2.
(3) **Completely random chains, no monomer**. In case of random chains (*p* = 1) in complete absence of all monomers (meaning *M* = 0), the random walk may initially resemble the result of case 1 (pure monomer). However, with an increasing number of random walks over time, the statistical result of the corresponding sequences will reflect the given sequences of the chains. Even though they have formed randomly, they will determine the total statistics of an infinite set of random walks over time. Therefore, an algorithm that is meant to reproduce these statistics must contain the sequence of every single given chain, together with a subroutine deciding on the random decisions on where to start and where to connect from chain to chain. This means that its code necessarily contains all sequences and hence is determined either by *c*_1_ in Equation (5) or by *c*_2_ in Equation (6), depending on which result is smaller. Some additional code is required for the hopping between chains. In any case, the result will be significantly larger than in cases 1 and 2. The system may have formed randomly, nevertheless its state is quite complex. In Figure 1, this situation would correspond to point 3.
(4) **Completely defined chains, no monomer**. In a system without any residual monomer (which means that *M* = 0) and fully defined chains with a given sequence (*p* = 0), the random walk will follow the defined sequence (or parts of it) repeatedly. The starting unit and the connecting positions between the chains are the only random points of the pathway. Correspondingly, the reproducing algorithm would have to include the defined sequence of the chain together with an occasional call for the random number generator. Hence, the number of bit or byte necessary for this algorithm depends on the length *L* of the defined chain and is slightly larger than *c* = *L* ∙ log_2_ *n* (in bit). This system’s degree of complexity may be smaller than in case 3 (since *N* is generally larger than *L*), but it definitely exceeds that of the cases 1 or 2 (point 4 in Figure 1).

## 5. Model Calculations

In order to show some characteristic results, model calculations for order according to Equation (3) and complexity according to Equations (9) and (10) have been performed showing some important dependencies. All calculations are for four different monomer types (*n* = 4, like in RNA) and an overall amount of 1 mol units (0.25 mol for each base). It turns out that, for a system size of one mol, *c*_1_ is always smaller than *c*_2_, such that only Equation (9) is relevant for the degree of complexity. Due to the macroscopic size of the system, the complexity can vary over many orders of magnitude up to the Tbit regime, while the order (given as the reciprocal entropy) varies between 0.1 and 0.3 K/J. The variations of the entropy are thermodynamically relevant and can correspond to the effect of several kJ of heat at room temperature.

**Figure 3:**
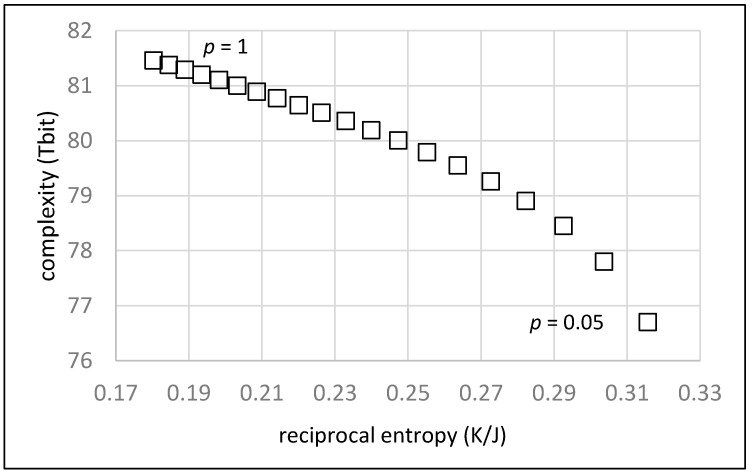
Order/complexity diagram showing the effect of an increasing fraction of defined chains (shown as a decreasing fraction *p* of random chains, from left to right in steps of 0.05).

Figure 3 shows the dependence of the position in an order/complexity diagram vs. the fraction *p* of random chains. The value for *p* is varied between 1.00 and 0.05 in steps of 0.05. An average chain length of *L* = 20 units and an average number of *M* = 10 monomers between two chains on the random walk are assumed.

As expected, the order of the system given as the reciprocal entropy continuously increases with an increasing fraction of defined chains (decreasing *p*). At the same time, the complexity decreases, since there are less and less random chains to be accounted for. The overall result in Figure 3 clearly shows that a simple increase of the fraction of defined chains does not advance evolution as indicated by the diagonal in Figure 1. The system may increase its degree of order, but simultaneously loses complexity such that the potential for further development is reduced.

**Figure 4:**
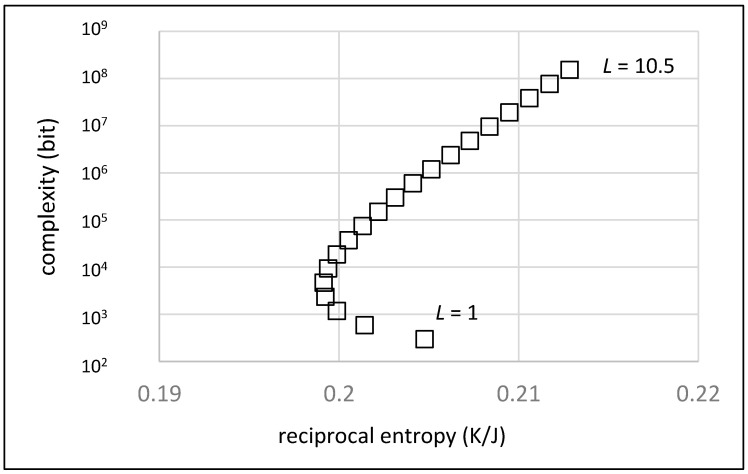
Order/complexity diagram representing the effect of increasing average chain length (from bottom to top in steps of 0.5 units). Due to the large increase of the system’s complexity, this parameter is shown on a logarithmic scale.

The effect of increasing average chain length *L* on the order/complexity diagram is shown in Figure 4. The value for *L* varies between 1 (meaning that there are just monomers and no chains at all) and 10.5 in steps of 0.5. An average fraction of random chains of *p* = 0.5 and an average number of *M* = 10 monomers between two chains on the random walk are assumed.

As expected, the complexity increases dramatically with increasing chain length, the values spanning over six orders of magnitude. Regarding the order, the dependence is more complex. Initially, for chain lengths between 1 and 5 units, reciprocal entropy decreases with increasing numbers of connected units due to the increasing number of system components. At this point, the mixing entropy dominates. With longer chains (*L* > 5), order increases again since more and more units are connected. Comparing the overall development with the evolution diagonal (Figure 1), it shows that only the development of longer chains can be seen as a true contribution to a successful evolution process provided that at least a fraction of the chains do have a defined sequence.

**Figure 5:**
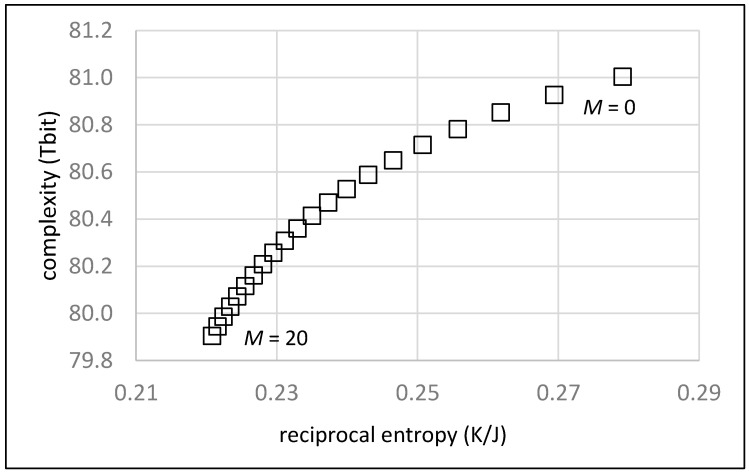
Order/complexity diagram showing the effect of decreasing average number of monomer units between chains on the “random walk” (from left to right). The value of M = 0 corresponds to a situation without any monomer.

Figure 5 shows the effect of a decreasing number of monomer units *M* on the path of the “random walk”. In this sense, the value for *M* varies from 20 to 0 in steps of 1. An average chain length of *L* = 20 units and an average fraction of random chains of *p* = 0.5 are assumed.

The resulting development shows a continuous increase in order and complexity and almost ideally follows the evolution diagonal of Figure 1. An increasing contribution of the oligomer chains vs. the monomer units basically stands for a more efficient polymerization capability and generally for a more evolved system.

**Figure 6:**
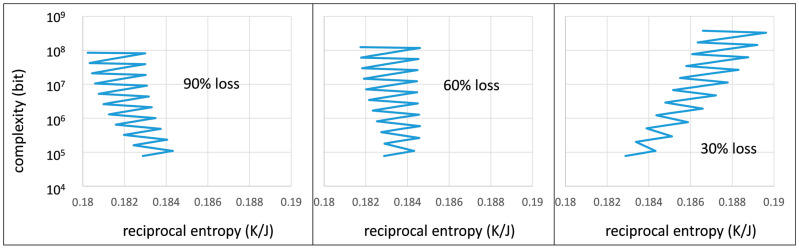
Simulated evolution process over 50 time units (from bottom to top) following a simple mutation/selection scheme and for different partial loss of defined chains during the mutation events: 90% (**left**), 50% (**center**), 30% (**right**).

What could an actual evolution process look like? In nature, it could consist of random changes (e.g., mutations) followed by periods of selection. In the following model calculations (Figure 6), we assume a constant selection rate given by d*p*/d*t* = −0.01 per time unit interrupted by mutation events in regular time intervals (every five time units). During each mutation event, a certain percentage of the defined chains is lost, while a single unit adds to the length of the defined chain. We also assume that the defined chains are, on average, twice as long as the random chains. The result of this simplified evolution model strongly depends on the degree of loss of defined chains during the mutation event. With the periodicity of the mutation/selection events, each curve follows a zigzag pattern, but the overall development leads to different final results after 50 time intervals.

At 90% loss (Figure 6, left), the development leads to a more complex situation, but obviously to a significant decrease in structural order. The destructive influence of the mutation events is not balanced out by selection, but still leads to increasing system complexity. The situation at 60% loss during the mutation event seems more or less static in terms of the system’s order state. However, at 30% loss of chain definition, the selection process obviously gains ground over time and leads to a stepwise increase of order and complexity. Such a pattern can be seen as a typical development for an evolution process. Regarding the structural order that develops during these steps, one can see this development as the outcome of a continuous learning process [26,27,28,34]. In this context, the spontaneous reduction of the sequential entropy, which in a thermodynamic sense derives from energy dissipation e.g., connected to base pairing, can be interpreted as the result of the second law of learning [26].

The self-organization process leading to an increase of order is typically interpreted as a simple consequence of reduplication and selection. Stuart A. Kauffman has offered a significantly broader view on self-organization [35]. According to this generalized model, catalytic and regulatory networks will form among macromolecules such that they reproduce in a cooperative manner, a mechanism that is strikingly similar to functional patterns found in organisms [35]. A system of competing networks can be seen as a more complex contribution to the selection process leading to an increasing degree of structural order, here simply represented by every second step in the zig-zag course in Figure 6.

**Figure 7:**
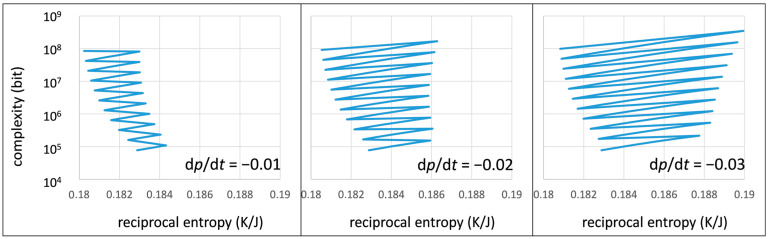
Simulated evolution process over 50 time units (from bottom to top) following a simple mutation/selection scheme and for different selection rates d*p*/d*t*: −0.01 (**left**), −0.02 (**center**), −0.03 (**right**).

The negative outcome at a loss rate of 90% at each mutation step (Figure 6 left) can be partially compensated by a higher selection efficiency (increasing from left to right in Figure 7).

With increasing selection rate (given by a negative development of the random chain fraction), the system may lose more order (reciprocal entropy) during each mutation step. On the other hand, each following selection process becomes more and more efficient since the selection starts with more complex material to choose from. Overall, this can end up in increasing values for order and complexity at the end of each selection period (circles in Figure 7 right). In the long run, however, an evolution process with a less destructive mutation step is much more efficient (Figure 6 right). Only a continuous development towards increasing order and increasing complexity is suitable for an efficient step towards initial forms of life.

## 6. Summary and Outlook

Based on the idea of an RNA world, the concept of order and complexity proves to be a versatile and powerful approach to evaluate the efficiency of general evolution processes. It defines a general criterion for any development in any system that presumably is capable to form functional prebiotic chemistry and to transform into early forms of life. Beyond the application shown here, and with minor additions, it easily accounts for special features such as multilayer and membrane environments, micelle and vesicle formation, intermolecular arrangements, compartment formation, surface interactions, temperature and pressure gradients. Further, it is suitable as a very general tool to identify life or any early stages of its development and yields a thermodynamic understanding for its various states of order.

## Data Availability

Not applicable.

## Supplementary Materials

The following supporting information can be downloaded at: https://www.mdpi.com/article/10.3390/life13030603/s1, File S1: Justification of Equation (1).
