# Supplementary material for: Order and Complexity in the RNA World
Source: Life (Basel). 2023 Feb 21;13(3):603. doi: 10.3390/life13030603 (PMC10052177; doi:10.3390/life13030603)
Supplement: Supplementary file 1 [file life-13-00603-s001.zip › life-2202700-supplementary.pdf]

File S1: Justification of Eq. [1]

A) General approach for a sequential entropy

In statistical thermodynamics, the term entropy is defined according to the Boltzmann formula

$$S \quad = \quad \mathrm{k} \ln w \qquad \text{[S1]}$$

with k as Boltzmann's constant and w as the statistical weight of the system's state. We assume that a system consists of N separate particles of different varieties i = 1, 2, 3, …n. If each variety i occurs at a number of m_i (with $m_1 + m_2 + m_3 + \ldots + m_n = N$), the number of different sequences obtained in a random walk through all of the particles (and hence the corresponding statistical weight of the system's state) is given by:

$$w \quad = \quad \frac{N!}{m_1! \times m_2! \times m_3! \times \ldots. \times m_n!} \qquad \text{[S2]}$$

Or, in a logarithmic form:

$$\ln w = \ln N! - \ln m_1! - \ln m_2! - \ln m_3! - \ldots - \ln m_n! \qquad \text{[S3]}$$

With the approximation $\ln x! \approx x \ln x - x$ for large numbers x and accounting for $m_1 + m_2 + m_3 + \ldots + m_n = N$, we get:

$$\begin{aligned} \ln w \quad &= \quad N \ln N \ - \ \textstyle\sum_{i=1}^{n} m_i \ln \mathrm{m}_i \\ &= \quad \textstyle\sum_{i=1}^{n} (m_i \ln N - \ m_i \ln \mathrm{m}_i) \end{aligned} \qquad \text{[S4]}$$

When we introduce the fraction $p_i = m_i / N$ of each variety i, this expression turns into:

$$\begin{aligned} \ln w \quad &= \quad -N \quad \textstyle\sum_{i=1}^{n} p_i \ln p_i \\ &= \quad N \quad \textstyle\sum_{i=1}^{n} p_i \ln (1/p_i) \end{aligned} \qquad \text{[S5]}$$

Together with the Boltzmann formula Eq. [S1], this leads to a term for the sequential entropy:

$$S \quad = \quad \mathrm{k}\, N \quad \textstyle\sum_{i=1}^{n} p_i \ln (1/p_i) \qquad \text{[S6]}$$

This result is completely analogous to the thermodynamic entropy of N particles with n different states $i = 1, 2, 3, \ldots n$ and given populations p_i where $p_1 + p_2 + p_3 + \ldots + p_n = 1$.

In a system that consists of only one single type of units (that is, $p_1 = 1$ while all other populations are zero), all random walks would yield identical sequences. According to Eq. [S6], this leads to $S = 0$. In a system that consists of e.g. four different units with equal populations $p_i = ¼$, the corresponding result is $S = \mathrm{k}N \ln 4$.

B) A characteristic random walk

We now need to consider an averaged characteristic random walk through the system following the rules listed in the text. Assuming that the system consists of interconnected units forming chains of an averaged length L as well as of monomer units in solution, we assume the random walk to connect an average of M free dissolved units before it connects to another chain. The contact from the unit M onto the next chain preferably occurs at a chain position with a high accessibility a_i. Starting from this point, the path follows the chain in any direction over a certain number of chain units until the end of the chain, where the path (for M > 0) regularly continues towards the dissolved units such that the described step starts from the beginning. The overall average number of chain units is necessarily determined by $L/2$. So on average, a typical step including one path through the solution and one stretch of a chain includes $M + L/2$ units. Considering a random walk over N units, we need to account for $N/(M + L/2)$ repetitions of this step.

During each such step (one path through the solution and one stretch of a chain), three different contributions to the sequential entropy have to be accounted for: i) the path through the monomers in solution, ii) the contact point between the last monomer unit and the chain, iii) the path along the chain to one of its end points. All these contributions are considered in the following.

1) Path through the monomer solution

This contribution to the sequential entropy is determined by the number of monomer units per step M and by the relative populations r_i of the n different monomer types where $r_1 + r_2 + r_3 + \ldots + r_n = 1$. In analogy to Eq. [S6] and for $N/(M + L/2)$ steps, it reads:

$$S_1 \quad = \quad \mathrm{k}\, M\left(\frac{N}{M+\frac{L}{2}}\right) \quad \sum_{i=1}^{n} r_i \ln\left(1/r_i\right) \qquad \text{[S7]}$$

For just one single monomer type and $r_1 = 1$, this contribution is zero. For e.g. four different monomer types with equal populations, it turns into $(\ln 4)\ \mathrm{k}\, MN/(M + L/2)$.

2) Contact point between last monomer and first chain unit

The second contribution to the sequential entropy is determined by the relative accessibility a_j of the L different chain units where $a_1 + a_2 + a_3 + \ldots + a_L = 1$. In analogy to Eq. [S6] and for $N/(M + L/2)$ steps, it reads:

$$S_2 \quad = \quad \mathrm{k}\left(\frac{N}{M+\frac{L}{2}}\right)\sum_{j=1}^{L} a_j \ln\left(1/\mathrm{a}_j\right) \qquad [S8]$$

For just one single accessible unit j in the chain and $a_j = 1$, this contribution is zero. For equal accessibility of all chain units, it turns into (ln L) k $N/(M + L/2)$.

3) Path along the chain

The third contribution to the sequential entropy is determined by the relative predictability p_k of each following unit in the chain with $p_1 + p_2 + p_3 + \ldots + p_n = 1$. If the chain sequence is completely determined, all predictability values are set to $p_1 = 1$ with the index $k = 1$ denoting the most likely unit type. If the chain is completely random, all predictability values are equal to $1/n$. In analogy to Eq. [S6] and for $L/2$ units in each of the $N/(M + L/2)$ steps, it reads:

$$S_3 \quad = \quad \mathrm{k}\,\frac{L}{2}\left(\frac{N}{M+\frac{L}{2}}\right)\sum_{k=1}^{n} p_k \ln\left(1/p_k\right) \qquad [S9]$$

For a completely determined sequence with $p_1 = 1$, this contribution is zero. For a completely random chain consisting of e.g. four different units, it turns into (ln 4) k $(L/2)N/(M + L/2)$.

In combination, all three contributions S_1, S_2 and S_3 add up to Eq. [1] in the main text. If N is set to $6.022 \cdot 10^{23}$, the resulting value $S_r = S_1 + S_2 + S_3$ accounts for a molar sequential entropy with respect to the average unit. Its thermodynamic equivalent is the mixing entropy. This mixing entropy would determine the driving force for the complete loss of a fully determined sequence.
